# Temporal causal inference with stochastic audiovisual sequences

**DOI:** 10.1371/journal.pone.0183776

**Published:** 2017-09-08

**Authors:** Shannon M. Locke, Michael S. Landy

**Affiliations:** 1 Dept. of Psychology, New York University, New York, NY, United States of America; 2 Center for Neural Science, New York University, New York, NY, United States of America; Radboud Universiteit, NETHERLANDS

## Abstract

Integration of sensory information across multiple senses is most likely to occur when signals are spatiotemporally coupled. Yet, recent research on audiovisual rate discrimination indicates that random sequences of light flashes and auditory clicks are integrated optimally regardless of temporal correlation. This may be due to 1) temporal averaging rendering temporal cues less effective; 2) difficulty extracting causal-inference cues from rapidly presented stimuli; or 3) task demands prompting integration without concern for the spatiotemporal relationship between the signals. We conducted a rate-discrimination task (Exp 1), using slower, more random sequences than previous studies, and a separate causal-judgement task (Exp 2). Unisensory and multisensory rate-discrimination thresholds were measured in Exp 1 to assess the effects of temporal correlation and spatial congruence on integration. The performance of most subjects was indistinguishable from optimal for spatiotemporally coupled stimuli, and generally sub-optimal in other conditions, suggesting observers used a multisensory mechanism that is sensitive to both temporal and spatial causal-inference cues. In Exp 2, subjects reported whether temporally uncorrelated (but spatially co-located) sequences were perceived as sharing a common source. A unified percept was affected by click-flash pattern similarity and the maximum temporal offset between individual clicks and flashes, but not on the proportion of synchronous click-flash pairs. A simulation analysis revealed that the stimulus-generation algorithms of previous studies is likely responsible for the observed integration of temporally independent sequences. By combining results from Exps 1 and 2, we found better rate-discrimination performance for sequences that are more likely to be integrated than those that are not. Our results support the principle that multisensory stimuli are optimally integrated when spatiotemporally coupled, and provide insight into the temporal features used for coupling in causal inference.

## Introduction

Multisensory interactions are beneficial to the observer as they can facilitate detection in noisy environments, improve accuracy of perceptual judgements, and allow for faster reactions to sensory events [1]. For example, imagine encountering a rattlesnake on a walk. Combining a brief glimpse of movement with an audible rattling noise will increase the probability that you notice the snake and correctly localise where it is hiding, as well as reducing the time to turn around and run. However, it is also important not to confuse sources of sensory information with different origins, such as the snake’s rattle and a nearby crawling insect. Determining whether sensory information should be integrated based on a shared origin is referred to as casual inference [2]. Temporal proximity is a well studied causal-inference cue that is used in solving this correspondence problem. Intuitively, sensory events that occur together in time are more likely to have originated from a common source.

The importance of temporal synchrony in multisensory integration has been extensively demonstrated in the audiovisual domain. Physiological results demonstrate that multisensory neurons in the superior colliculus are sensitive to temporal disparity between audiovisual signals, with small disparities producing weaker responses and larger lags causing depression from baseline firing [3]. Neuro-imaging has revealed that temporally coincident audiovisual signals increase activity in the multisensory superior temporal sulcus, which in turn modulates activity in the primary sensory cortices [4]. Furthermore, behavioural experiments have demonstrated temporally sensitive multisensory enhancement effects between audition and vision for single brief events [5], as well as longer, dynamic sequences of events [6–10].

The brain, however, is not a perfect judge of when something occurred [11]. This stems in part from the differences in the speed of light and sound, which causes concurrent audiovisual signals to reach the observer with some distance-dependent inter-sensory latency. Additionally, the speed of transduction differs between the senses; the cochlea is a much faster transducer than the retina. Numerous studies have shown that when light leads sound by some tens of milliseconds, the stimuli are more likely to be perceived as synchronous [12] and judged as coming from a common source [13]. Whereas, signals with other temporal disparities may still be combined but with lower probability as long as they are within the “temporal binding window” [14, 15]. This window represents a trade-off between too little and too much integration, selecting only concurrent multisensory signals for binding yet being invariant to inter-sensory delays. The size of this audiovisual window is variable across subjects and perceptual tasks [16, 17]. For example, observers judge brief auditory clicks and light flashes to be synchronous with disparities up to approximately 100 ms [18] yet are able to combine complex audiovisual speech with disparities of up to 200 ms [19].

However, recent studies indicate that the role of temporal correspondence in multisensory integration may not be a general phenomenon, but rather depend on the task at hand. Observers integrate temporally independent click-flash sequences when estimating rate [20, 21] but not when judging location [6]. Raposo et al. [20] suggested that the insensitivity to temporal correspondence in rate discrimination might be the result of a different kind of multisensory integration mechanism than the canonical circuits investigated previously [1, 3], one that is not concerned with the relative timing of sensory inputs. Instead of being preserved, information about the timing of events across modalities is discarded in rate estimation as this task requires monitoring the density of events within a temporal window substantially broader than that used to judge temporal coincidence. In such a scenario, observers are presumably relying on other casual-inference cues, such as spatial proximity, to judge whether the sources should be integrated [2, 5, 22].

A second hypothesis offered by Raposo et al. [20] was that the rates used, 7 to 15 events/s, were too fast to determine temporal correspondence. In contrast, Parise et al. [6], in a localisation task, used random audiovisual sequences with an event rate of 5 events/s. For periodic pulse trains faster than 4 Hz, audiovisual synchrony discrimination is at chance [23], and integration occurs regardless of phase [8]. The temporal frequency of 4 Hz is also the lower limit for auditory driving [24, 25], where the auditory flutter rate dramatically influences the perceived visual flicker rate of a visual stimulus. However, the ability to determine audiovisual temporal correspondence may also rely on stimulus attributes other than the sensory event rate. For example, mouth opening and auditory envelope are two well-known cues in speech perception, but are typically modulated at 2 to 7 Hz in natural speech [26]. Speech is a temporally complex signal compared to the simple rhythmic stimuli used to measure the 4 Hz limit for synchrony perception. Indeed, when click-flash sequences are made more complex by increasing the randomness of inter-event intervals, observers are more accurate at reporting temporal correspondence [27]. The stimuli in the localisation task [6] were more random in terms of inter-event intervals and therefore more complex than those used in the rate-discrimination task [20], where inter-event intervals were always either 60 or 120 ms. This may have further increased the chances of subjects detecting temporal correspondence in the localisation task, even though stimulus rates were above 4 events/s. In sum, observers may use temporally sensitive multisensory integration mechanisms for both tasks, but are unable to gauge the synchrony of the sequences in the rate-discrimination task due to fast event rates and low stimulus complexity.

A third plausible hypothesis is that observers are able to integrate separate rate estimates without any regard for the probability the sources share a common origin. This flexible coupling of information across the auditory and visual senses has been demonstrated previously. For example, observers combine visual dots and sounds moving in opposite directions to better detect motion [28]. In another study, subjects flexibly coupled rotational dot motion and a sound burst according to task instructions [29]. This form of integration is driven exclusively by task demands, rather than the spatiotemporal nature of the stimuli.

**Fig 1:**
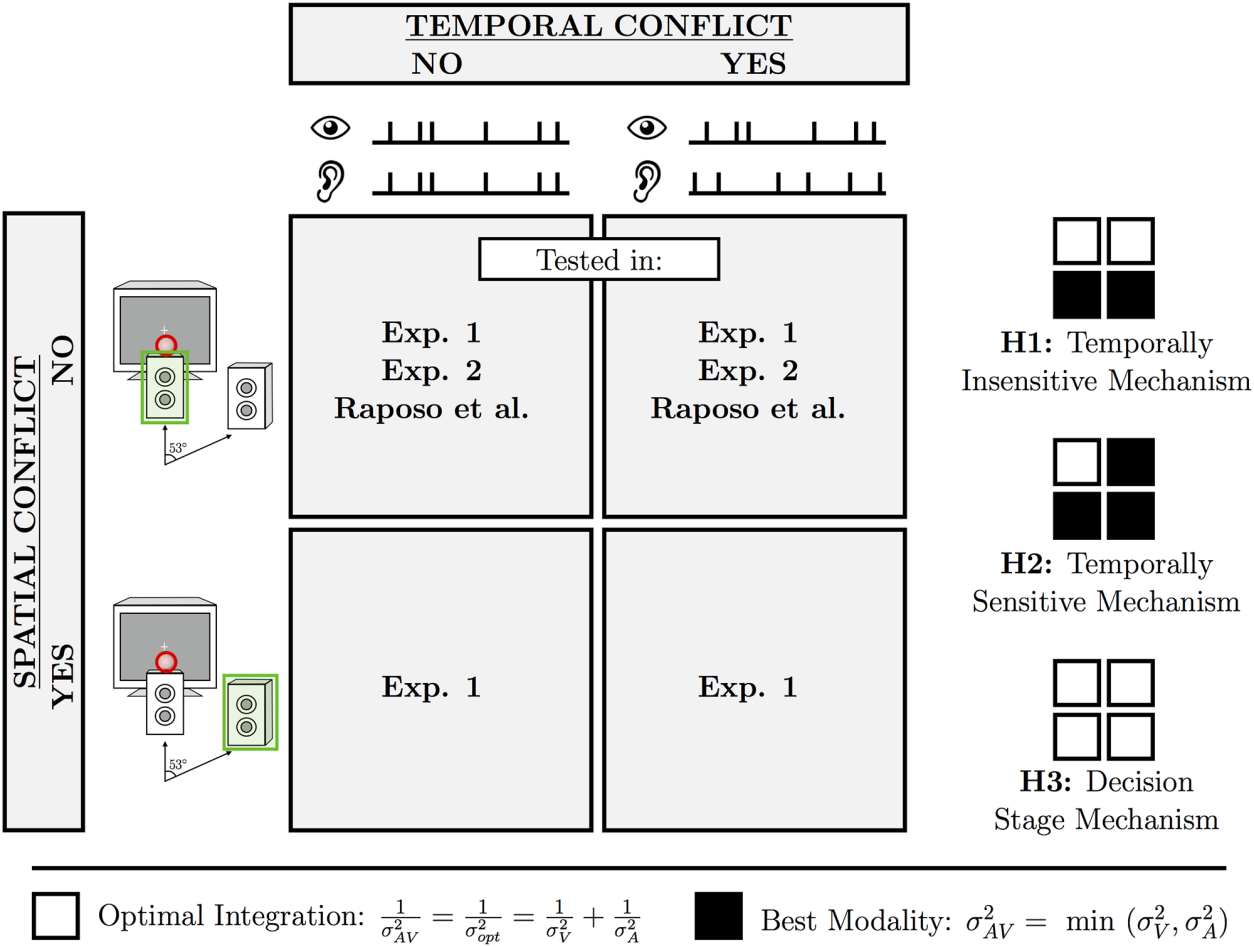
Design and hypotheses of Exp 1. The four spatiotemporal conflict conditions were defined by the spatiotemporal relationship between the auditory and visual signals in Exp 1. Only temporal conflict was examined in Exp 2 and the previous rate-discrimination study of Raposo et al. [20]. The small grids on the right show the predicted pattern of results in Exp 1 under the four hypotheses.

Here we present the results of two experiments. Experiment 1 investigated how humans integrate audiovisual signals for rate estimation to test whether the multisensory integration mechanism is indeed insensitive to temporal correspondence. Importantly, our stimuli were presented at slower rates and with greater temporal complexity than the previous rate-discrimination task [20] to increase the chance of finding temporal sensitivity. Rate-discrimination performance was assessed in four conditions: audiovisual signals could have temporal conflict, spatial conflict, spatiotemporal conflict, or no conflict. Each of the three hypotheses outlined above gave a distinct pattern of predictions (Fig 1). If the multisensory mechanism is temporally insensitive but spatially sensitive, subjects will rely solely on spatial cues to determine correspondence and integrate only in the no-conflict and temporal-conflict conditions (Hypothesis 1). Alternatively, if subjects are able to determine temporal correspondence with our slower, more complex stimuli, then they should only optimally integrate in the no-conflict condition if they are also spatially sensitive (Hypothesis 2) or in both the no-conflict and spatial-conflict conditions if they are spatially insensitive (Hypothesis 3). Finally, if subjects simply couple information because of task demands, regardless of the spatiotemporal relationship, then they should show multisensory integration in all four conditions (Hypothesis 4).

Reflected in our hypotheses above are different outcomes of spatial conflict, which are not the focus of this study but bear mentioning. Seminal studies conducted in the superior colliculus of the cat demonstrated that multisensory enhancement was diminished by spatially separating the signals [30, 31]. This led to the “spatial rule” of multisensory processing, stating that spatial overlap, at least at the level of neural receptive fields, promotes integration. However, the necessity of spatial congruence has since come into question, and appears to play less of a role in temporal judgements unless spatial attention is key to the task [32]. In relation to this study, there is some evidence in temporal multisensory tasks that spatial congruency is not necessary for the perception of numerosity [33] or rate [34, 35]. Thus the results of Exp 1 will also be relevant to this discussion on the applicability of the spatial rule.

In Experiment 2 we looked beyond rate discrimination to investigate more broadly the temporal causal-inference cues available from stochastic audiovisual sequences using causality judgements. The salience of three temporal features were examined: the proportion of synchronous click-flash pairs, the maximum offset between any sequential click and flash, and overall temporal pattern similarity. To relate the results of Exp 1 to Exp 2, we took two approaches. First, we compared simulations of the sequence-generating algorithms of Exp 1 and the previous rate-discrimination study [20] in terms of both the relative presence of the three sequence features of interest and their saliency in causal inference. And second, we examined whether there were observable differences in rate discrimination in the presence of temporal conflict, based on whether it was more likely the participant perceived the auditory and visual sequences as sharing a common origin or separate origins.

## Experiment 1

### Methods

#### Participants

Ten participants took part in both Exp 1 and 2 (3 male, age 22–34), including the author SML. An additional subject completed Exp 1, but was excluded from analysis due to difficulty with the task (results outside the bounds of the adaptive procedure) and was not asked to complete Exp 2. All participants had normal or corrected-to-normal vision and no known hearing problems. This study was approved by the New York University Committee on Activities Involving Human Subjects. In accordance with the ethics requirements of the Institutional Review Board at New York University, participants received details of the experimental procedures and gave informed consent prior to the experiment.

#### Setup

Experiments were conducted in a darkened sound-attenuating booth offering up to 35 dB of attenuation. Visual stimuli were presented on a Dell 2209WA LCD monitor (51.3 x 36.3 cm, 60 Hz refresh rate). A chin rest was used to fix the subject’s head position at a distance of approximately 37 cm. Auditory stimuli were presented via Advent AV570 speakers at a 48 kHz sampling rate. The central speaker was spatially aligned with the midline, and a lateral speaker 53 deg to the right of midline. The experiment was run using custom written MATLAB (version R2014a, MathWorks) software, with the Psychophysics toolbox [36–38] used for stimulus presentation. All responses were made on a standard computer keyboard.

#### Task

Exp 1 examined the effect of spatial and temporal conflict on audiovisual rate discrimination by measuring behaviour under four conditions: no conflict, spatial conflict, temporal conflict, and spatiotemporal conflict (Fig 1). A single random sequence was used in conditions without temporal conflict, and two independent sequences were generated for those with temporal conflict (one for clicks and one for flashes). Spatial conflict trials used the speaker that laterally displaced the auditory signal from the visual signal.

**Fig 2:**
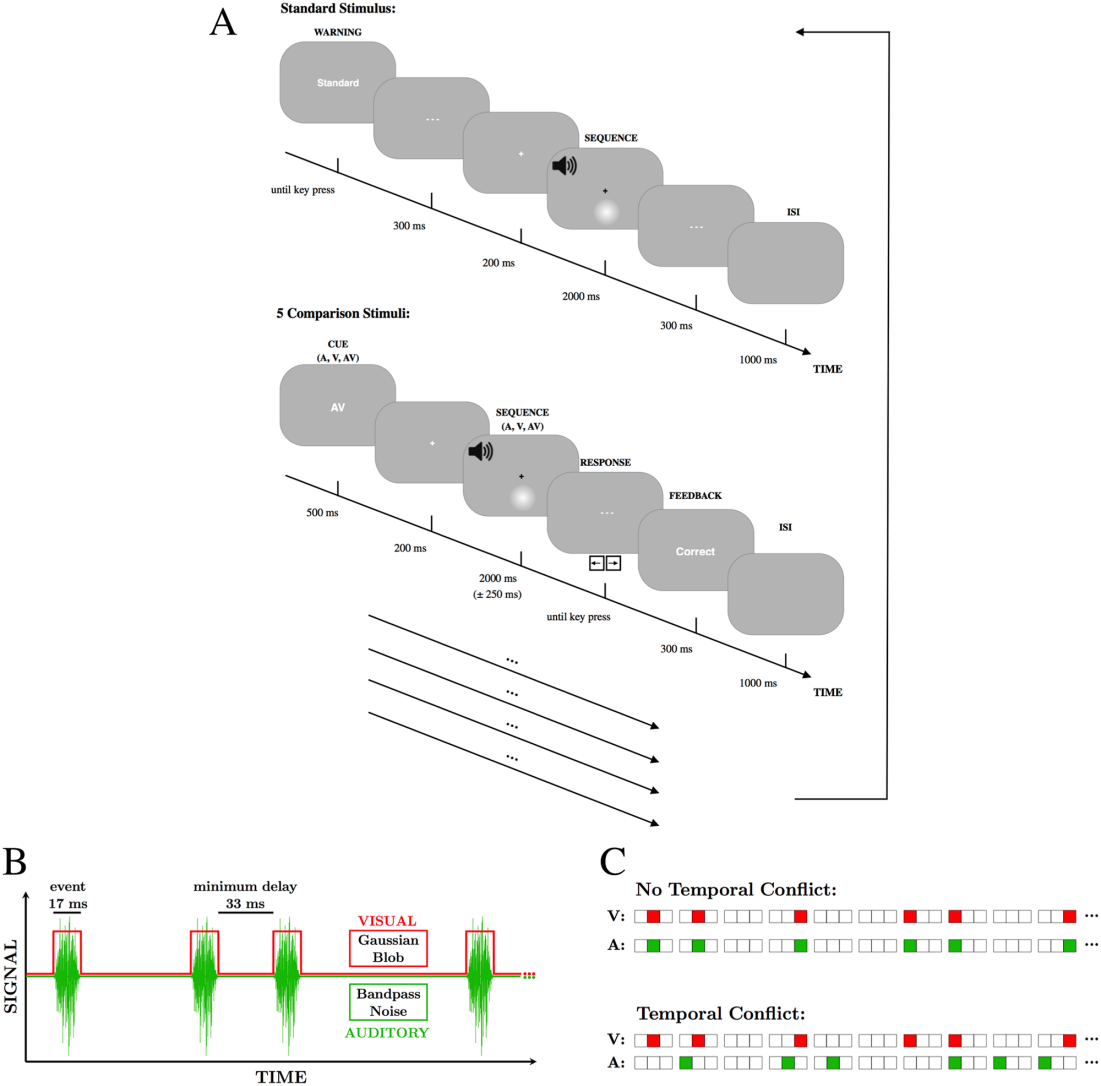
Task and stimuli of Exp 1. (A) Time course of standard and comparison stimulus presentations. A multisensory standard stimulus was passively viewed before every five comparison stimulus trials (one shown in full, the remaining indicated by the additional arrows). For each trial, the modality of the comparison stimulus was pseudorandomly selected to be auditory, visual, or audiovisual. Participants indicated whether the comparison stimulus was faster or slower than the standard. (B) Portion of an example synchronous sequence. Visual stimulus (red) follows a square wave modulation pattern and the auditory stimulus (green) is a cosine-ramped bandpass signal. (C) Sequence-generating algorithm. Frames are divided into triplets, and events are assigned within the triplet randomly unless the previous triplet contains event, in which case events are in the identical position as in the previous triplet.

Participants completed a comparison-to-standard rate-discrimination task with randomly interleaved visual, auditory, and audiovisual trials. Each conflict condition was examined in a separate session, the order of which was randomised across participants. In the task participants were required to judge if the comparison stimulus was faster or slower than an 8 events/s standard stimulus. In each trial, the comparison stimulus was presented at a rate between 2–14 events/s, and could be auditory, visual, or audiovisual. The standard stimulus was always audiovisual and matched the conflict condition of the session. The standard stimulus presentation was only every five trials and was preceded by a textual cue and required no response from the participant. Before every comparison stimulus, the subject received a textual cue (‘V’, ‘A’, or ‘AV’) informing them of the modality or modalities in which the stimulus would subsequently be presented. Response feedback was given at the end of each trial. See Fig 2A for more details.

Before each session, participants completed a training block of 150 trials mixed across the three modes of presentation (visual, auditory, or multisensory). In the main task, three separate adaptive procedures with 200 trials each were completed for each presentation mode. We modified an adaptive procedure from version 2.2 of the UML toolbox [39] for this experiment. This procedure estimates the psychometric function trial by trial using all available stimulus-response pairs collected and chooses sampling points along the psychometric function that lead to parameter estimates with the lowest variance (see S1 File for more details).

While the use of an adaptive procedure allowed us to rapidly collect the data for twelve psychometric functions per participant, it precluded the use of the unobserved category boundary paradigm used by Raposo et al. [20]. This is because the average rate of the comparison trials was not guaranteed to be equal to the 8 events/s standard, as would have been the case using the method of constant stimuli. Instead, we presented the standard every five trials and provided feedback to encourage use of the correct category boundary. Furthermore, preference was given to collecting both unisensory and multisensory thresholds for a conflict condition in a single session, so as to avoid spreading the trials of the adaptive procedure over consecutive days. The effect of fluctuations in discrimination performance due to the order of sessions (learning) or testing on different days was minimised by only comparing unisensory and multisensory thresholds collected within the same session. One advantage of this method is that participants were always aware of where the stimuli were going to be presented and could direct their spatial attention accordingly. However, this method doesn’t allow us to address strategy-switching or sensory adaptation concerns.

#### Stimuli

A visual event involved a brief flash of a luminance-defined Gaussian blob (SD of 2.5°) in the peripheral visual field, with the centre of the disk located 7.9° below the fixation cross. The disks were white on a uniform mid-grey background (50% contrast). Auditory events were bandpass noise bursts (200 Hz–10 kHz) with a 5 ms cosine ramp applied to sound onset and offset (Fig 2B). The sound level was adjusted to be comfortable and clearly audible for each subject. Easily detectable visual and auditory stimuli were used so that detection failures would not reduce the rate estimate.

Each stimulus presentation was a stream of visual and/or auditory events. The standard stimulus was always 2000 ms long; comparison stimuli had ±250 ms of random duration jitter (in units of 3 frames or 50 ms) to discourage counting. The beginning and end of each presentation interval was indicated in both the visual and auditory modalities. The fixation cross was white for 200 ms prior to trial onset, it switched to black until the end of the stimulus, after which the cross disappeared (Fig 2A). A short 16.7 ms 900 Hz tone played immediately before and after the presentation interval. Participants were instructed to consider the rate across the entire interval defined by these start and end markers.

To generate an event sequence, the number of events was calculated based on the rate and stimulus duration. Events were then randomly designated to time bins of length 3 frames (50 ms). Within a frame triplet, an event was 1 frame in duration (16.7 ms, for both visual and auditory events) and the other 2 frames were blank/silent (Fig 2C). The event frame within the triplet was chosen randomly with one exception: if an event occurred in the preceding frame triplet, the event must occupy the same position in the current triplet. This ensured a minimum delay of 33 ms between events. The sequence-generation algorithm was used once for trials with no temporal conflict, and twice for temporal-conflict trials to create independent sequences. In trials with spatial conflict, the auditory stimuli were presented from the laterally displaced speaker, and in no-spatial-conflict trials the auditory stimuli were presented from the centre speaker immediately below the visual stimulus.

#### Calculation of thresholds

Cumulative Gaussian psychometric functions describing the relationship between proportion of “comparison faster” judgements and difference in rate between the comparison and standard stimuli were fit individually for each subject, session, and modality (120 total). Parameter estimates, mean *μ* and variance *σ*, and 95% confidence intervals were obtained with a custom-written MCMC algorithm in R version 3.2.1, using RStan version 2.8.0 [40]. Flat priors were used for both parameters, with the ranges determined from a pilot study: *μ* ∼ U(−7, 7) and log(*σ*) ∼ U(−2.3, 1.6). Marginal posterior distributions for each parameter were approximated from 2000 MCMC samples. The *μ* parameter is the point of subjective equality (PSE), i.e., the rate difference for which the subject is equally likely to respond that the comparison stimulus was faster or slower than the standard. The *σ* parameter reflects sensitivity to differences in rate, with smaller values indicating greater sensitivity. We will refer to this parameter as the sensitivity threshold for rate discrimination.

In our analysis, we considered two possible models multisensory behaviour, the best-cue strategy and optimal cue integration. The best-cue strategy involves selecting the more reliable modality and using information only from that signal to estimate rate. With *σ*_*A*_ and *σ*_*V*_ representing the auditory and visual thresholds respectively, the multisensory threshold under the best-cue strategy is

$$\begin{array}{c} \sigma_{best}^{2}=\text{min}\left(\sigma_{V}^{2},\sigma_{A}^{2}\right). \end{array}$$ (1)

Optimal cue integration involved weighting the information from each modality according to its relative reliability, to produce a multisensory estimate with the highest reliability possible. The posterior distribution of the predicted optimal thresholds, *σ*_opt_, was computed on a per session basis for each subject. The posterior for the optimal threshold was approximated by taking independent draws, *s*, from the posterior distributions calculated in the unisensory threshold conditions, and computing for each drawn pair the optimal multisensory threshold *σ*_opt_ according to

$$\begin{array}{c} \frac{1}{\sigma_{\text{opt(s)}}^{2}}=\frac{1}{\sigma_{\text{A(s)}}^{2}}+\frac{1}{\sigma_{\text{V(s)}}^{2}}. \end{array}$$ (2)

The estimate of *σ*_opt_ and the 95% CI were calculated from these samples as described above.

#### Group-level analyses

Group-level analyses were performed to measure 1) the cost of switching attention between different modalities, and 2) the effect of jittering the duration of the stimuli. For these analyses it was not feasible to pool the raw data due to the considerable variation in the psychometric functions across participants, conditions, and modalities. To remove these additional sources of variability we z-transformed the raw data. This was achieved by using the parameters fitted in the individual analysis to transform the stimulus levels so that all psychometeric functions had *μ* of 0, and *σ* of 1. For subject *i* in condition *j*, we transform the *k*th stimulus level *r*_*ijk*_ as

$$\begin{array}{c} z\left(r_{ijm}\right)=\frac{r_{ijk}-\mu_{ijk}}{\sigma_{ijk}}, \end{array}$$ (3)

where *μ*_*ijk*_ and *σ*_*ijk*_ are the parameters of the fitted psychometric function from the individual-level analysis. After pooling all stimulus-response pairs (24000 trials total), they could be separated into different categories based on the particular analysis. For the attention-switching analysis, the pooled data were split into two categories: 1) the previous trial was in the same modality condition, or 2) the previous trial was of a different modality condition (including switches from unisensory to multisensory or vice versa). In the duration analysis, trials were sorted into short- and long-duration groups, which corresponded to duration jitter values of −250– −100 ms and 100–250 ms, respectively (omitting trials with smaller values of jitter). A second duration analysis was performed on each of the 11 jittered durations, which were spaced at 50 ms (three frame) intervals.

#### Bayesian model comparison

For each conflict condition, we computed whether the optimal-integration model or the best-cue model best describes the observers’ behaviour using a Bayesian model comparison. The Statistical Parametric Mapping software package version 12 [41] was used for this analysis. Using a hierarchical Bayesian method described by Stephan et al. [41], we estimate the probability of each model at a group level by treating the model as a random variable and fitting the parameters of a Dirichlet distribution over all models. This method is superior to reporting information-criterion scores as it both reflects a robust group statistic and is adept at handling inter-subject variability. Results are reported in terms of exceedance probabilities, describing how much more likely one model is than the other.

Model evidence was computed separately for each participant-condition pairing using the predicted thresholds described in Eqs 1 and 2 and a fixed PSE value. In the best-cue model we used the fitted PSE of the more reliable modality: *μ* = *μ*_*best*_. In the optimal-integration model we used *μ* = 0. We chose an unbiased PSE for the optimal model in lieu of any clear theoretical alternatives. As Exp. 1 was not a cue-conflict study, construction of a theoretical prediction would require an explanation for any observed PSE biases, which could be due to a variety of factors that may or may not differ between the unisensory and multisensory conditions. If this zero-bias assumption is incorrect, the optimal model will be handicapped in favour of the best-cue model, because model evidence is reduced when it isn’t calculated at the best-fitting parameter settings. In the case that the less reliable cue is selected for the judgement, this unbiased PSE may favour the optimal model if the signs of the PSE bias are opposite for the two unisensory cases. We believe that this is an insignificant problem as all cases of worse-than-best-cue behaviour were restricted to conditions where the best-cue model wins.

### Results

**Fig 3:**
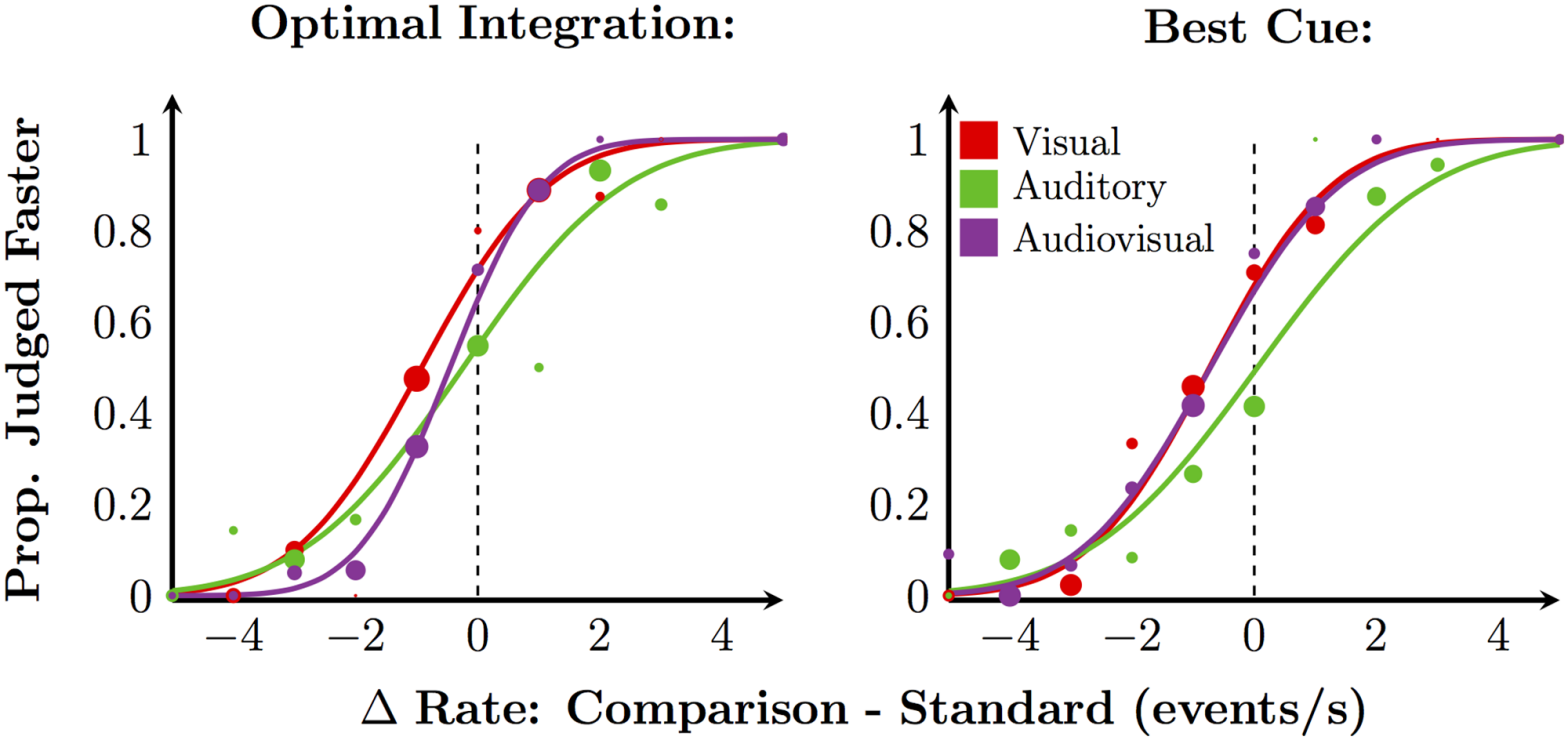
Example psychometric functions from Exp 1. Psychometric functions of an example observer showing the behaviour consistent with the optimal cue-combination strategy (left panel) and, in another condition, with the best-cue strategy (right panel). Solid curves: fitted functions. Markers: binned raw data. Marker diameter is proportional to the log of the number of trials in that bin.

We measured rate-discrimination performance in four conditions defined by the spatiotemporal relationship between the auditory and visual sequences. To estimate rate-sensitivity thresholds, cumulative Gaussians were fit to the data describing the probability of perceiving the comparison stimulus as faster than the 8 event/s standard stimulus as a function of the rate of the comparison stimulus. The effect of conflict was measured by the relative magnitudes of the multisensory thresholds. If no integration occurs, the multisensory threshold should be equivalent to the best unisensory threshold (i.e., the best-cue strategy, Eq 1). If the observer combines the auditory and visual information by appropriately weighting the unisensory estimates by their relative reliabilities, then the threshold should be equal to the predicted optimal threshold (Eq 2). Fig 3 shows psychometric functions of a representative subject consistent with these two strategies. In the left panel, the audiovisual curve is steeper than both unisensory curves, indicating that both sources of information were used. In the right panel, the fit audiovisual curve overlaps the steeper of the two unisensory curves, i.e., the best cue was used; the two cues were not integrated. Thresholds higher than optimal but less than the best unisensory threshold are indicative of integration with sub-optimal weights on each modality.

**Fig 4:**
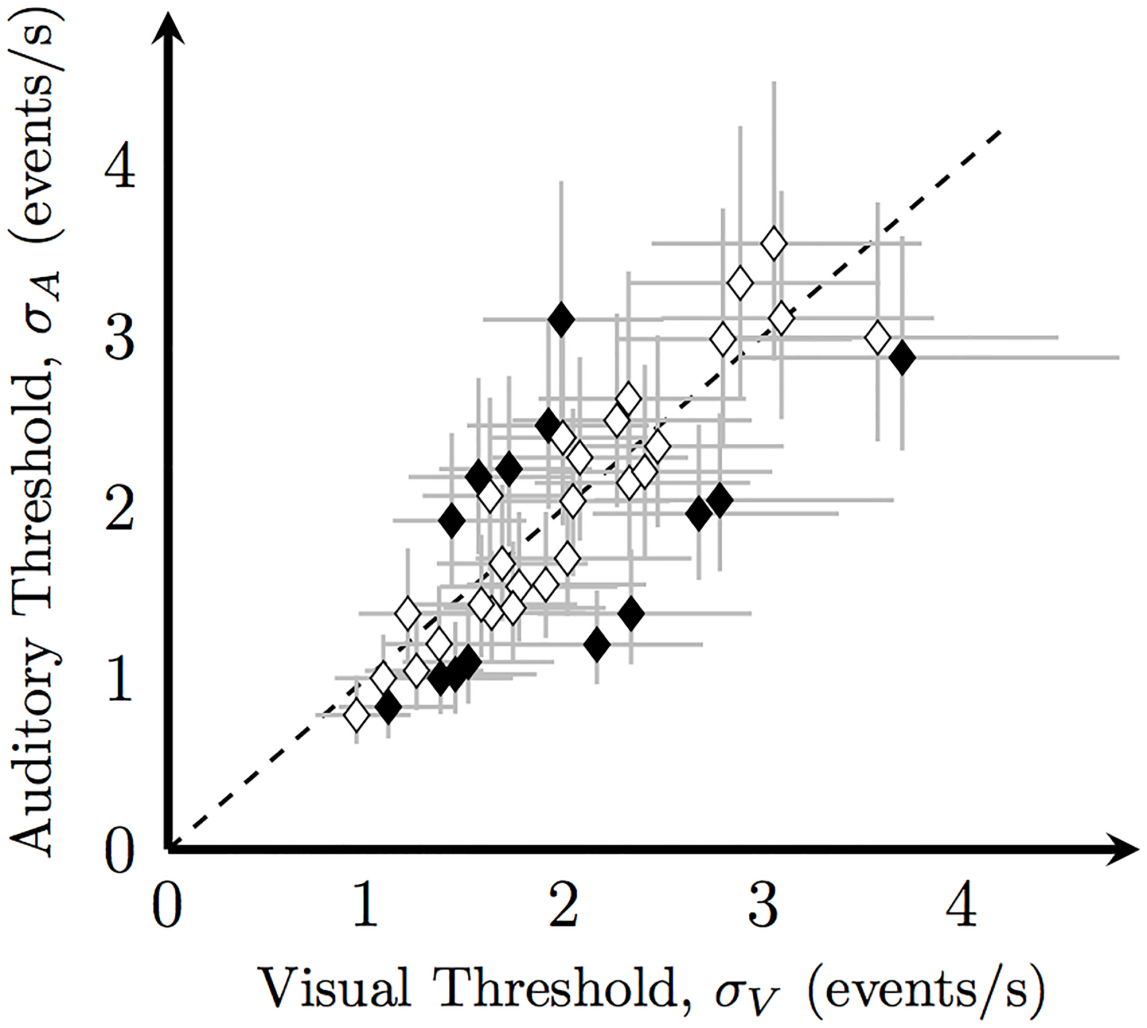
Comparison of the unisensory thresholds. Each data point represents a unisensory threshold pair computed for a single observer in one condition. Black fill indicates that the unisensory thresholds differ significantly. Error bars: 95% CIs.

The difference in thresholds between the best-cue strategy and optimal cue-combination strategy is maximal when unisensory thresholds are equal [42]. In multisensory experiments, unisensory performance is typically matched by degrading the quality of the more reliable signal. However, in the rate-discrimination task missed events lead to bias rather than higher rate-discrimination threshold. We used suprathreshold stimuli that were easily detectable to avoid this bias, but this did not allow us to match reliability across modalities. It has previously been shown that audition is more reliable than vision in rate estimation [34]. In our study, however, a paired t-test revealed no significant overall difference between the auditory and visual thresholds (Fig 4, *t*_39_ = 0.97, *p* = 0.34), indicating that our optimal-integration versus best-cue strategy comparison was appropriate.

**Fig 5:**
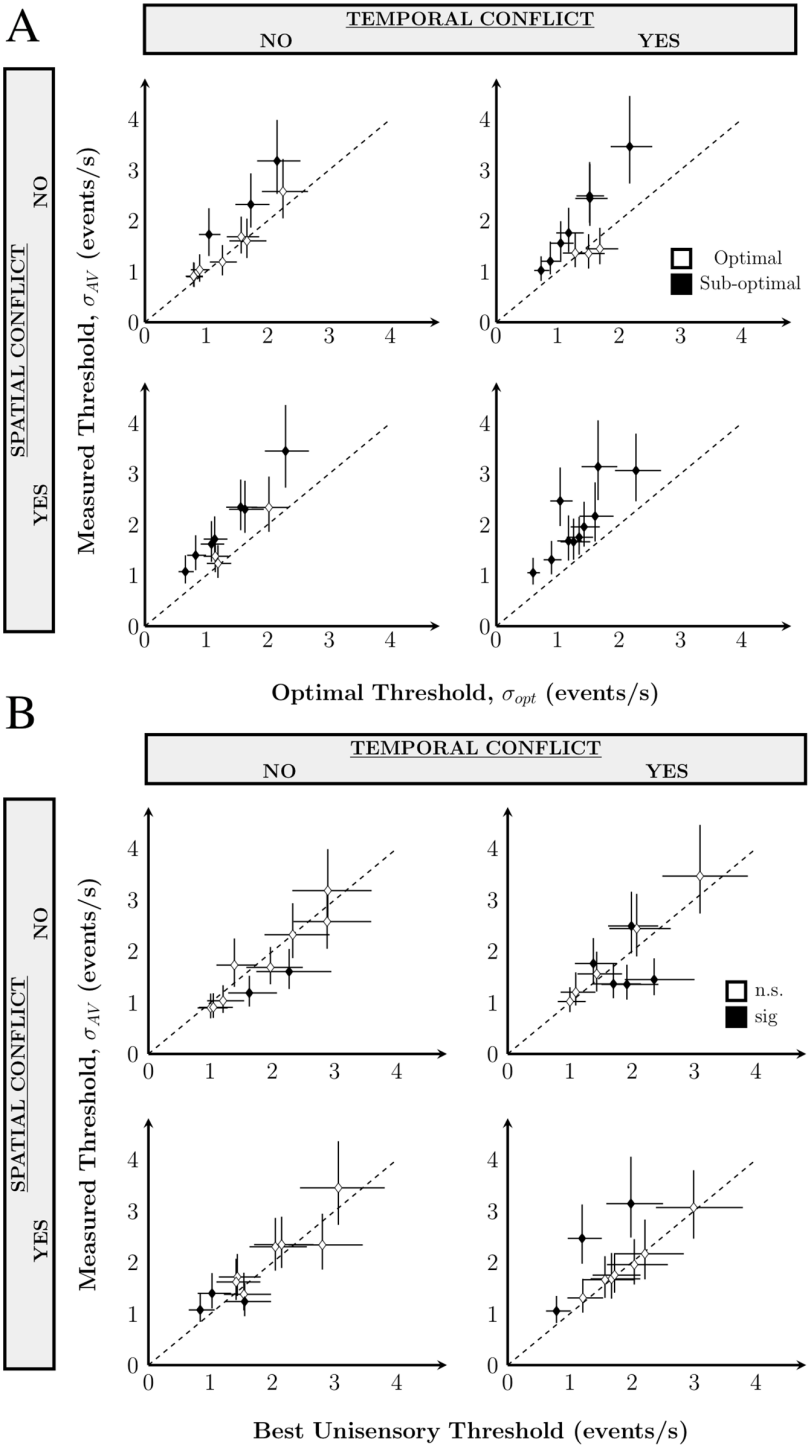
Thresholds in Exp 1. A: Measured multisensory threshold as a function of the threshold predicted for optimal integration based on the unisensory thresholds for each observer in each condition. Data points above the line (black) indicate that threshold was sub-optimal, whereas points along the equality line (white) indicate optimal integration. B: A comparison of measured thresholds with the best unisensory threshold. Points not along the equality line (black) indicate that the best-cue strategy was not used. Below the equality line indicates a multisensory enhancement effect and points above suggest poor cue selection strategy (i.e. using the less informative unisensory cue). Error bars: 95% CI.

Thresholds for audiovisual rate-dicrimination were compared to the optimal-observer predictions (see Methods). In the condition with no conflict, the majority of participants were indistinguishable from optimal. In contrast, most subjects in the conflict conditions were suboptimal (Fig 5A). Three out of ten subjects were indistinguishable from optimal in the spatial- and temporal-conflict conditions. A multisensory enhancement effect is evident when multisensory thresholds are smaller than the best unisensory threshold. Fig 5B compares multisensory thresholds with the best unisensory threshold. Two subjects have a significant multisensory enhancement effect in the no-conflict condition, three in the temporal-conflict condition, one in the spatial-conflict condition, and none in the spatiotemporal-conflict condition. Some individuals even had thresholds significantly worse than the best unisensory threshold (two for temporal conflict, two for spatial conflict, and three for spatiotemporal conflict). These subjects may have used the less reliable cue in making their rate judgements. Optimal integration requires consideration of the relative reliability of the unisensory cues. Thus, observers may not have accurate estimates of unisensory cue reliability in this task.

**Fig 6:**
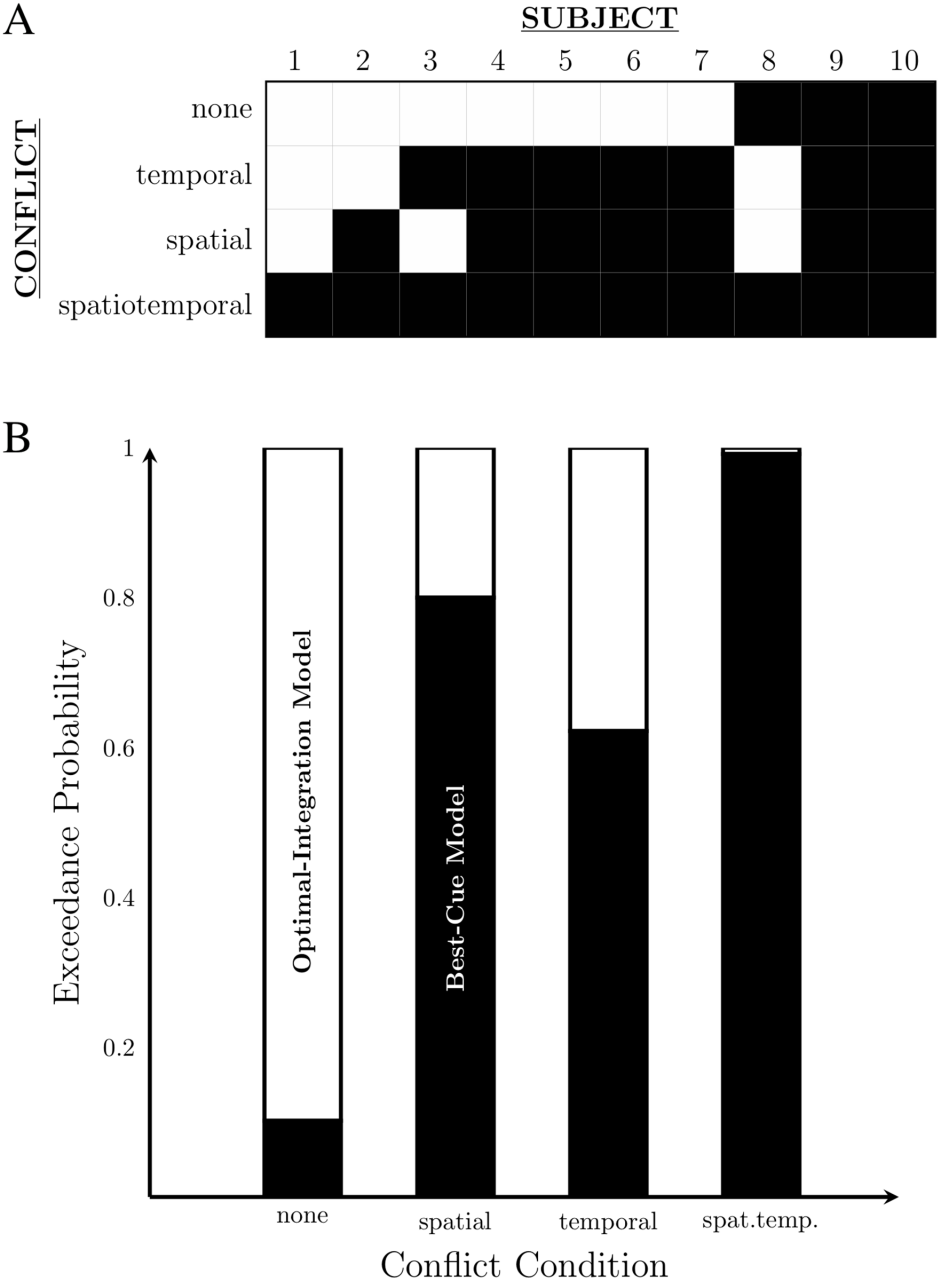
Optimal integration across subjects. A: Thresholds in the multisensory condition were classified as indistinguishable from optimal (white) or as suboptimal/no-integration (black). Predicted optimal thresholds were calculated from performance in unisensory trials. B: Exceedance probabilities from the Bayesian model comparison describing our belief that each model best describes the behaviour in the multisensory trials. White fill: optimal-integration model. Black fill: best-cue model.

Fig 6A shows the pattern of optimal integration for individual participants. High inter-subject variability is a common finding in multisensory experiments [43]. Thus, to determine which model (optimal integration or best-cue strategy) fit best at a group-level, we conducted a model comparison (see Methods). The exceedance probabilities for each spatiotemporal conflict condition show that optimal integration model only wins in the no-conflict condition, and the best-cue strategy wins in all of conditions with conflict (Fig 6B). Overall, these results support Hypothesis 2: observers use a temporally and spatially sensitive mechanism for audiovisual rate discrimination. It should be noted that the results presented in Fig 5 rely on the width of the posterior distribution, whereas the model-comparison outcome of Fig 6B was computed from the height of the likelihood function for specific parameter settings. Thus, while these two analyses are in agreement, the degree of support for each model differs slightly. In particular, the difficulty in observing deviations from the best-cue strategy due to the large CIs in Fig 5B was not an issue in the model-comparison analysis, which simply compared the degree to which the data supported each model.

**Fig 7:**
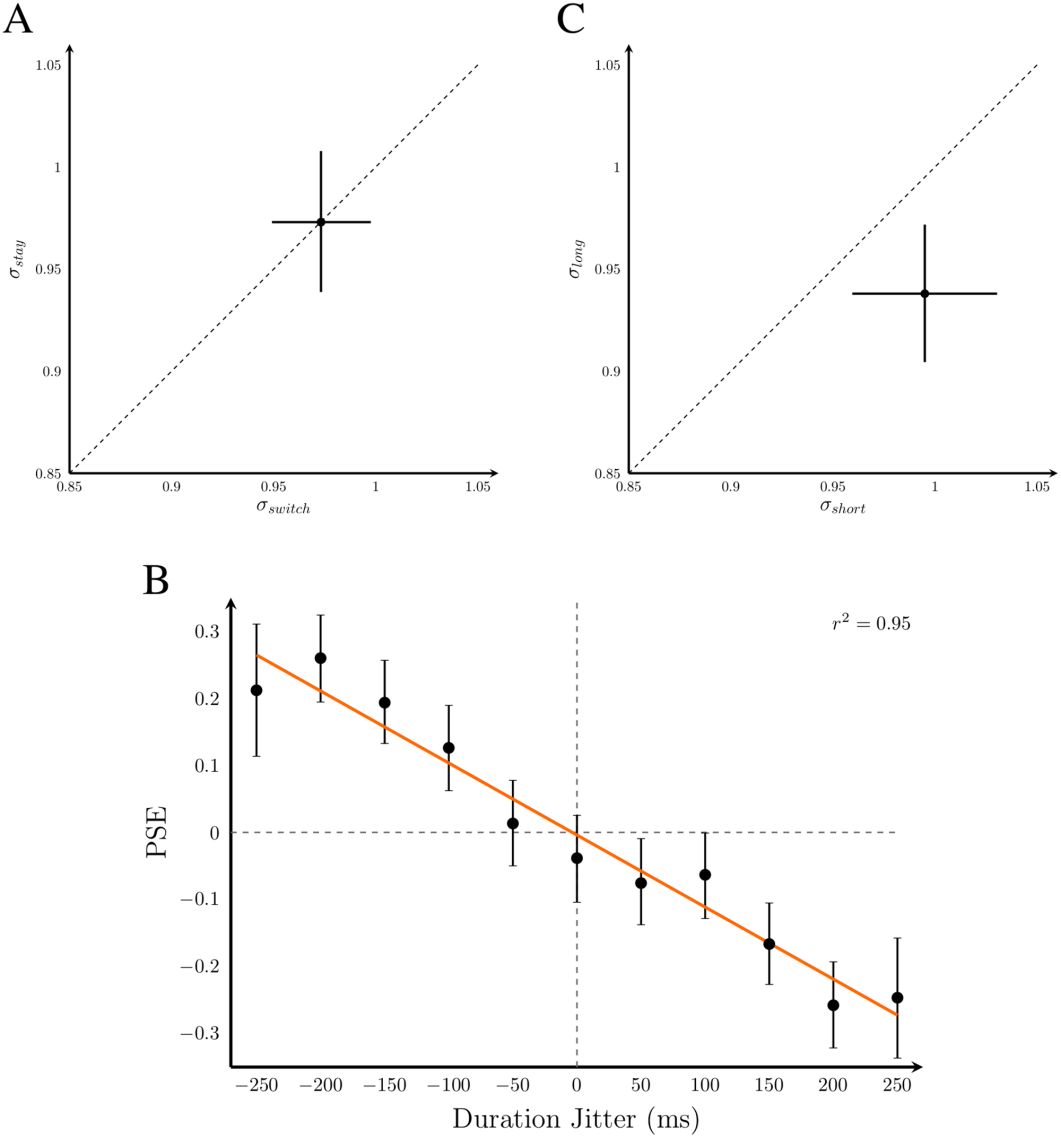
Effect of attention switching and trial duration on rate discrimination. Data for all plots were collapsed across subjects, presentation modality, and spatiotemporal conflict conditions. A: Rate sensitivity in the two attention-switching conditions: trials in same modality condition (stay) versus switching from one modality condition to another (switch). B: The measured point of subjective equality (PSE) for each comparison stimulus duration jitter. Orange line: linear fit to the data. C: Rate sensitivity for long trial durations (100 to 250 ms) versus short trial durations (−100 to −250 ms). Estimating differences in sensitivity requires more trials than differences in PSE, thus the data were split into short and long durations rather than assessed for each duration jitter value. PSE and sigma values are in units of SNR. Error bars: 95% CIs.

We next examined whether there is a cost in performance when switching from attending one modality to another, or in switching from unisensory to multisensory conditions, or vice versa. We did this by pooling data across subjects and sessions (see Methods), and comparing sensitivity in trials after a switch versus those in which the previous trial was from the same condition. As can be seen in Fig 7A, sensitivity did not differ significantly between switch and no-switch trials using a confidence-interval analysis. Thus, the modality cue presented before each trial was sufficient for subjects to orient their attention to the appropriate modality.

The second group-level analysis assessed whether subjects truly estimated rate, i.e., the density of events over time, or whether they instead estimated the total number of events. The results of Raposo et al. [20] indicate that subjects did not use a counting strategy, but this conclusion was based on a small number of trials. In comparison, our analysis involved all test trials presented, which varied in duration from the standard stimulus by up to 250 ms. The PSE had a strong negative correlation with trial duration (adjusted *r*^2^ = 0.95, *F*_1, 9_ = 199, *p* < 0.01). In this case, the PSE is the difference in rate required to perceive the comparison stimulus rate as identical to the 8 events/s standard stimulus, in units of signal-to-noise ratio (SNR) due to the *z*-transform (Fig 7B). For equal comparison and standard stimulus rates, observers are more likely to report “slower than the standard” if the comparison stimulus had a shorter duration and consequently fewer events. The opposite was true for trials with longer duration. This indicates that participants in our experiment were using a counting strategy; their judgements were influenced by the total number of events in the sequence.

We also found a significant difference in threshold between the short and long duration trials (Fig 7C). Longer durations resulted in lower thresholds, i.e., greater rate-discrimination accuracy. This improvement with longer duration is consistent with a density-estimation strategy in conjunction with evidence accumulation throughout the stimulus presentation [44]. Evidence accumulation in this scenario means that longer stimulus observations provided more sensory information and therefore led to more accurate rate judgements. Our finding is also consistent with the choice-triggered average analysis performed by Raposo et al. [20], where they concluded that information throughout the stimulus presentation affected the final rate estimate. Note that a strategy of estimating the count and then dividing by an estimate of stimulus duration, coupled with Weber’s law for both the count and duration estimates, would predict worse performance for long-duration trials, opposite to what we observed. Thus, there is evidence supporting both a counting strategy and a density-estimation strategy in Exp 1. However, due to the pooling technique used in this analysis, it is unclear whether particular stimulus levels, subjects, or conditions are responsible for this mixed-strategy result.

## Experiment 2

The results of Exp 1 show that humans can be sensitive to temporal conflict when estimating audiovisual rate. This suggests that the stimuli used in this experiment contained sufficient temporal information for causal inference, whereas the previous studies by Raposo et al. [20] and Sheppard et al. [21] did not. The aim of Exp 2 was to identify temporal features of stochastic audiovisual sequences that promote multisensory integration. Subjects performed a causality-judgement task, reporting whether they experienced a bound audiovisual percept or not. In this task, a bound percept for temporally independent sequences would indicate a failure to solve the temporal-correspondence problem [45]; an incorrect inference about the objective temporal structure of the stochastic audiovisual sequence. It should be noted that on a broader time scale of sequences these stimuli are temporally matched, which may be a compelling enough reason for observers to bind them despite their event-level mismatch. We considered: 1) the presence of these features in the stimuli of Exp 1 and the study of Raposo et al. [20] by simulating sequences with both stimulus-generation algorithms; and 2) whether these temporal causal-inference cues affected rate discrimination in Exp 1.

In the course of analysing the data of Exp 2, an experiment using a very similar paradigm was published by Parise & Ernst [13]. In this paper, the authors speculated that the optimal integration with temporally conflicting sequences observed by Raposo et al. [20] might be due to this causal-inference cue: “The reason may be that due to its low temporal resolution as a result of the low-pass filtering, the human perceptual system might simply become insensitive to the amount of correlation [between sequences] with increasing temporal rate.” (p. 5). Our study used faster rates (8 to 14 events/s compared to their 5 events/s). This allows us to directly assess in Exp 2 whether temporal pattern similarity as a temporal causal-inference cue can be generalised to faster event rates.

### Methods

#### Task and stimuli

All participants completed Exp 2 after Exp 1. The setup and sequence-generation algorithm were the same for both experiments. Stimuli in this task were always spatially congruent and presented in both modalities. Participants were presented with click-flash sequences with and without temporal conflict, and were asked to judge whether the clicks and flashes came from common or independent sources. The instructions encouraged them to think of the clicks and flashes as coming from mini explosions and to base their judgement on the entire sequence. Sequences were presented at four fast rates (8, 10, 12, and 14 events/s) for a fixed duration of 2 s, which ensured the same number of events were presented for each rate. Fast rates were used because they were more likely to be mistakenly perceived as synchronous than slower rates, and thus would be more informative about the salient temporal cues. To increase the probability of sequences with ambiguous temporal correspondence, sequences with an offset between subsequent click and flash exceeding 200 ms were discarded and a new sequence generated. No feedback was given in this experiment.

Each participant completed two 30 minute sessions on separate days, which always began with 25 practice trials. In each session there were 360 trials: for each rate there were 75 sequences with temporal conflict and 15 with no conflict. Subjects were informed that the number of stimuli from common and independent sources may not be equal, and to report their percept without taking into account their previous responses. Every 25 trials, participants were given a short break with an unrelated trivia game to keep them engaged and discourage tallying the frequency of their responses for each response option.

#### Simulated sequences

We simulated 1000 sequences with both the algorithm of Exp 1 and the algorithm of Raposo et al. [20] for each of the rates tested in Exp 2. Sequences at the slower rates of 4 and 6 events/s were also generated using the algorithm of Exp 1 to reflect the slower rates used in examining rate discrimination. Note that we did not impose on the simulated sequences the additional constraint of rejecting sequences with maximum click-flash offsets of 200 ms used in Exp 2. To reiterate the important constraints of both algorithms, in Experiment 1 each 3-frame interval was designated as event or no event by randomising the placement of events. The number of events was determined by the rate and duration. Within each frame triplet, the event frame was chosen randomly with one exception: If there was an event in the previous triplet, the next event was placed in the same frame within its triplet, ensuring a minimum 33 ms delay between events. In the study by Raposo et al. [20], inter-event intervals were either 60 or 120 ms. The relative number of 60 and 120 ms inter-event intervals was determined by the rate and their order randomised. Temporal-conflict trials also had a 20 ms offset between modalities to ensure events did not occur in both modalities at the same time.

### Results

Three sequence features were selected for analysis. The first was temporal pattern similarity (also assessed by Parise & Ernst [13]). For this feature, observers consider the match between the pattern of inter-event timing in one modality to the other modality. Precise timing of events between modalities is not crucial for this feature, so this cue allows for the known ambiguity in determining synchronicity between the senses, i.e., the temporal binding window. Greater pattern similarity should predict a higher probability of a “common source” judgement. The second cue we considered was the proportion of synchronous click-flash pairs. Human estimation of the relative timing of click-flash pairs is uncertain. Thus, the overall synchronicity of sequence could be a cue to temporal correspondence, where each click-flash pair could be treated as an additional observation in assessing overall probability of synchronicity. A higher proportion of synchronous pairs predicts a higher probability of a “common source” response. The final feature examined was the maximum offset between any consecutive click and flash. As in multisensory experiments with a single transient event, a large temporal offset signals a low likelihood of temporal correspondence, and would therefore be associated with a lower probability of a “common source” judgement.

In all analyses, only the temporal-conflict trials were used to fit the model; synchronous sequences did not contain useful variations in the features of interest. Across subjects, synchronous trials led to “common source” responses 97.1% of the time (±1.5% SEM). Temporal-conflict trials were identified as “common source” in 51.5% of trials (±5.8% SEM). This statistic confirms that temporal-conflict trials were sufficiently ambiguous in terms of the available temporal causal-inference cues.

**Fig 8:**
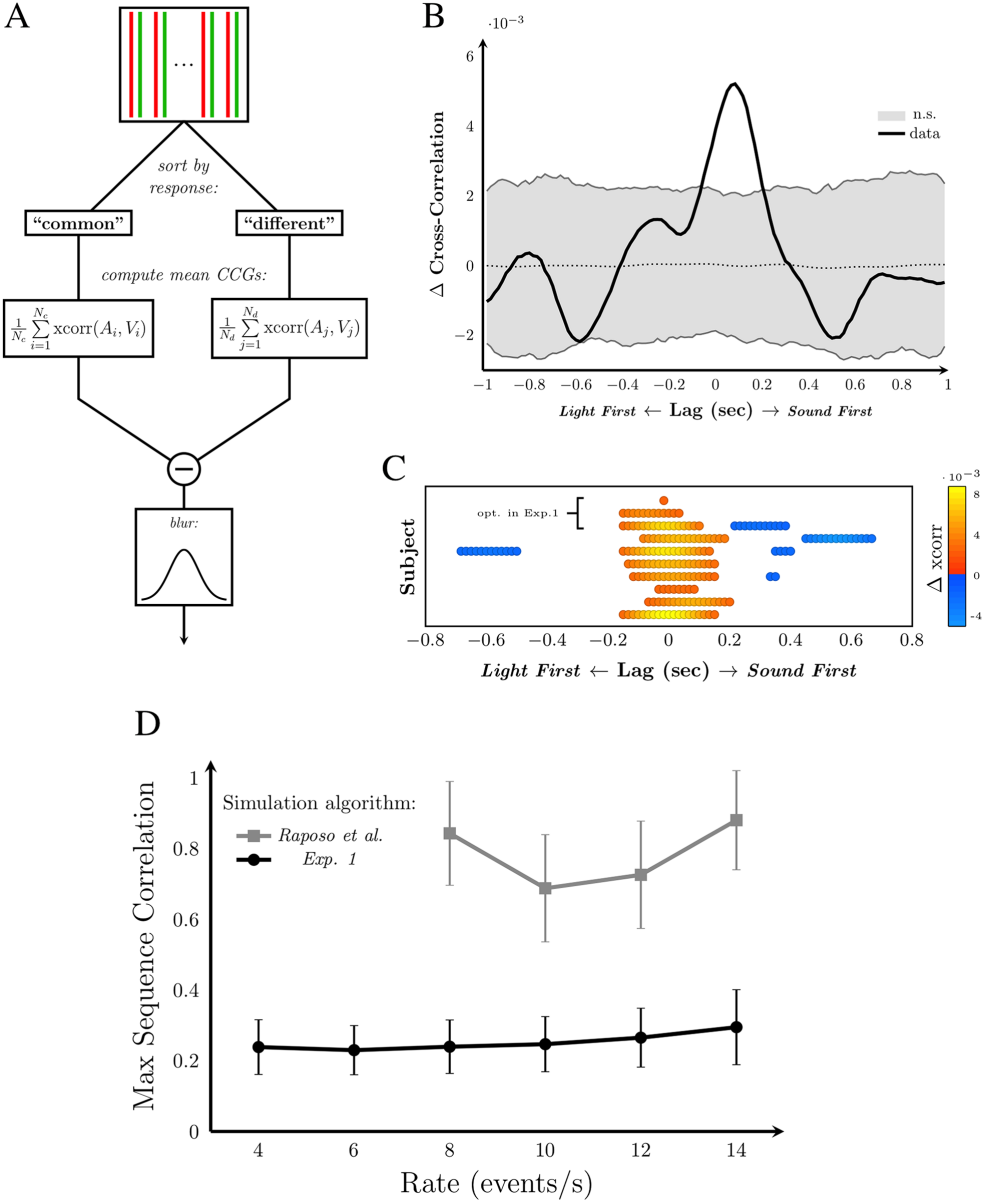
Pattern sensitivity in Exp 2. A: Procedure for deriving the cross-correlogram difference function, which was computed separately for each observer. Auditory and visual sequence pairs were sorted by the subject’s response in the causality-judgement task. Mean CCGs for each response were calculated, where *r*_*AV*_ was computed for audiovisual lags of −1 to 1 s and denoted as ‘xcorr(*A*, *V*)’ in the diagram. *N*_*c*_ and *N*_*d*_ refer to the number of “common source” and “difference source” judgements, respectively. The mean CCGs were then subtracted and smoothed with a Gaussian kernel with SD of 80 ms. B: The CCG difference function for an example subject. Grey region indicates non-significant differences, which were determined using a permutation test. As the mean CCG of “different source” was subtracted from the mean CCG of “common source”, positive values indicate correlations associated with “common source” judgements and negative values are associated with “different source” judgements. C: The significant regions of the CCG difference function for each subject. Orange indicates positive difference (associated with “common” responses). Blue indicates negative difference (associated with “different” responses). The three participants that were indistinguishable from optimal in the temporal-conflict condition of Exp 1 are indicated. D: The average maximum sequence correlation, within a ±200 ms temporal window, for the simulated sequences using the algorithms of Exp 1 and Raposo et al. [20]. Error bars: 1 SD.

A cross-correlation analysis was performed on an individual basis to assess whether temporal pattern similarity affected the response in the causality-judgement task. Cross-correlograms (CCG) describing the correlation between the auditory and visual sequence-pair were computed for each temporal-conflict trial. As introduced by Parise and Ernst [13], the average CCG was computed for each response type (“common” or “different”) and subtracted to produce a cross-correlation difference function (Fig 8A). We used a normalized cross-correlation value,

$$\begin{array}{c} r_{AV}\left(m\right)=(\frac{\vec{A_{m}}-E\left[\vec{A_{m}}\right]}{\left|\right|\vec{A_{m}}-E\left[\vec{A_{m}}\right]\left|\right|}\cdot \frac{\vec{V_{m}}-E\left[\vec{V_{m}}\right]}{\left|\right|\vec{V_{m}}-E\left[\vec{V_{m}}\right]\left|\right|}), \end{array}$$ (4)

resulting in correlation coefficient values between −1 and 1 for all temporal lags assessed. $\vec{A_{m}}$ and $\vec{V_{m}}$ refer to the overlapping portions of the auditory and visual signals for temporal shift *m*, respectively. The CCG difference function of an example subject is shown in Fig 8B. Positive values indicate greater correlation between the sequences for “common source” responses, whereas negative values indicate greater correlation for “different source” responses. Significant differences were determined using a permutation test. Response labels were shuffled and new CCG difference functions were computed 1000 times, resulting in 95% confidence boundaries under the null hypothesis that temporal pattern has no effect on the causality judgement. Any portion of the CCG difference function outside the confidence interval was considered to be a significant difference. The 95% confidence interval is indicated in Fig 8B by grey shading, and only the significant portions of each subject’s CCG difference function are plotted in Fig 8C.

All subjects had some significant portion of their CCG difference function, most commonly spread around zero lag where a higher correlation between the auditory and visual sequences is associated with “common source” responses. Consecutive significant data points are more indicative of wide temporal windows of temporal pattern sensitivity than scattered significant points, which would more likely represent Type I errors. There is also evidence of asymmetry in these temporal windows for some individuals, consistent with previous findings from single-event experiments that the optimal audiovisual temporal lag for audiovisual binding shows substantial individual differences and is often not centred at 0 [12]. Some subjects also showed regions with the opposite effect: correlations at particularly large temporal lags are associated with “different source” responses. This result suggests some subjects may have detected pattern similarity but concluded that the sequences did not share a common origin due to the large temporal offset. Such a result could not be found from single-event paradigms, and indicates that additional causal-inference cues are present when observers encounter dynamic sequences.

Fig 8C also shows that participants with performance indistinguishable from optimal in the temporal conflict condition of Exp 1 differ from one another in terms of their pattern-similarity sensitivity. The observer with only a single significant data point in the CCG difference function could be considered relatively insensitive to temporal pattern similarity, which may explain why this observer was able to combine audiovisual signals in temporal conflict. However, the other two optimal observers show a markedly different pattern of results, more closely resembling the observers who were not optimal performers in Exp 1.

In sum, these results indicate that temporal pattern similarity is a cue used in causal inference: high correlations at low lags increase the probability of concluding that the sequences share a common source, and correlations at larger lags can signal the opposite. Sequences with greater structure, like those of Raposo et al. [20], are more likely to have substantial correlations at small temporal lags than those that are more random as shown by our simulation analysis (Fig 8D). However, our results concerning the pattern-similarity cue did not distinguish observers with optimal versus sub-optimal performance. This suggests that other sequence features may contribute to the causal-inference judgement, to which we now turn.

The two other potential temporal-correspondence cues were assessed in a Generalised Linear Mixed-effects Model (GLMM) analysis. A logistic linking function was used because the dependent variable was binary (“common” or “different”). The fixed effects were 1) the proportion of synchronous click-flash pairs, 2) the maximum click-flash temporal offset, and 3) an intercept term. The random effect in the model was a subject identifier. We predicted a higher proportion of synchronous click-flash pairs would be associated with greater probability of a “common source” judgement, whereas a higher maximum click-flash offset would lead to a lower probability of a “common source” judgement. These two cues were found to have a significant weak correlation (*r* = −0.20, *p* < 0.01) according to a Pearson’s product-moment correlation test. As expected, both cues were also significantly correlated with the average rate of the sequence: *r* = −0.53 (*p* < 0.01) for maximum click-flash offset and *r* = 0.32 (*p* < 0.01) for proportion of synchronous click-flash pairs.

**Fig 9:**
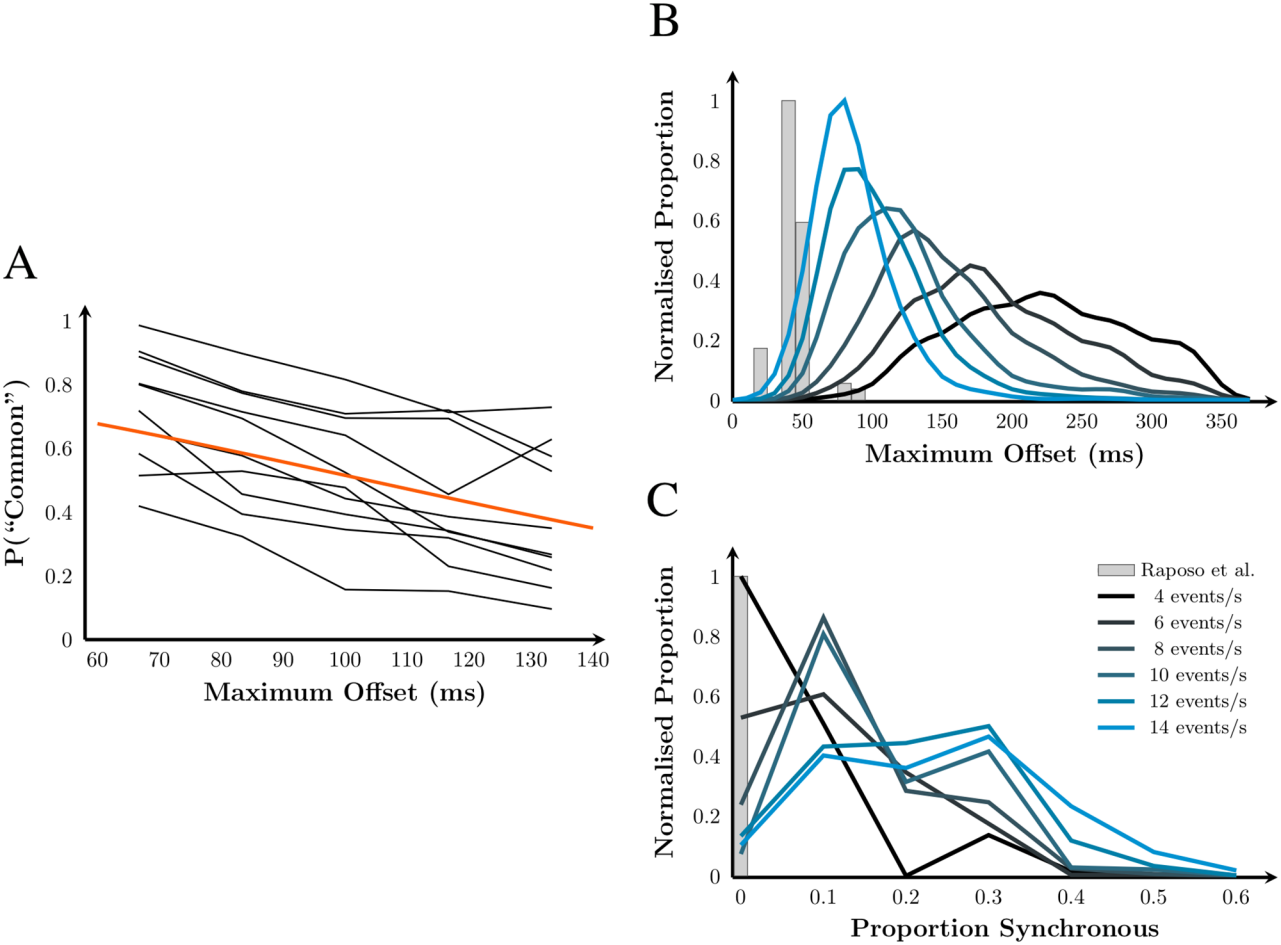
An analysis of temporal cues in Exp 2. A: Orange curve: The relationship between maximum click-flash offset and probability of a “common source” response as determined by the GLMM analysis. Black curves: The raw data for individual subjects, binned at 1 frame (16.7 ms) intervals, highlights inter-subject variability. Only the range of maximum offset shown had enough responses in Exp 2 to calculate the proportion of “common” judgements. B: Histograms of maximum temporal offset for each sequence-generating algorithm, normalised such that the maximum proportion was 1 for each simulation algorithm. Grey: histogram of simulated Raposo et al. [20] sequences, pooled across rates because the maximum offset was not affected by rate. Blue: density histograms for our sequence generating algorithm by rate (smoothed by a Gaussian kernel: SD 26.7 ms) C: Histograms of the proportion of synchronous click-flash pairs as a function of the generating algorithm and sequence rate.

The causality judgement was significantly affected by a main effect of maximum click-flash offset (*p* < 0.01), and no other main or interaction effect was found other than for the intercept term (*p* < 0.01). Significance was computed by parametric bootstrapping of the GLMM fit. The mean estimate and 90% confidence intervals for the maximum click-offset effect and the intercept term were −17.00 s (−19.70 s, −14.31 s) and 1.76 (1.22, 2.29), respectively. The fitted model and raw subject data are shown in Fig 9A. As predicted, the greater the maximum click-flash offset, the lower the probability of a “common source” judgement. This result is consistent with an effect of temporal conflict in Exp 1 and the absence of an effect in Raposo et al.’s experiment, according to our simulation analysis. As can be seen from the sequence statistics shown in Fig 9B and 9C, the algorithm used by Raposo et al. [20] eliminated the less salient cue of synchronous click-flash events, but at the cost of small click-flash offsets that are unlikely to signal a lack of temporal correspondence. Thus, observers in their task would be likely to integrate audiovisual signals despite the temporal conflict, whereas observers in Exp 1 are less likely to integrate because they are given stronger cues to accurately determine temporal correspondence.

**Fig 10:**
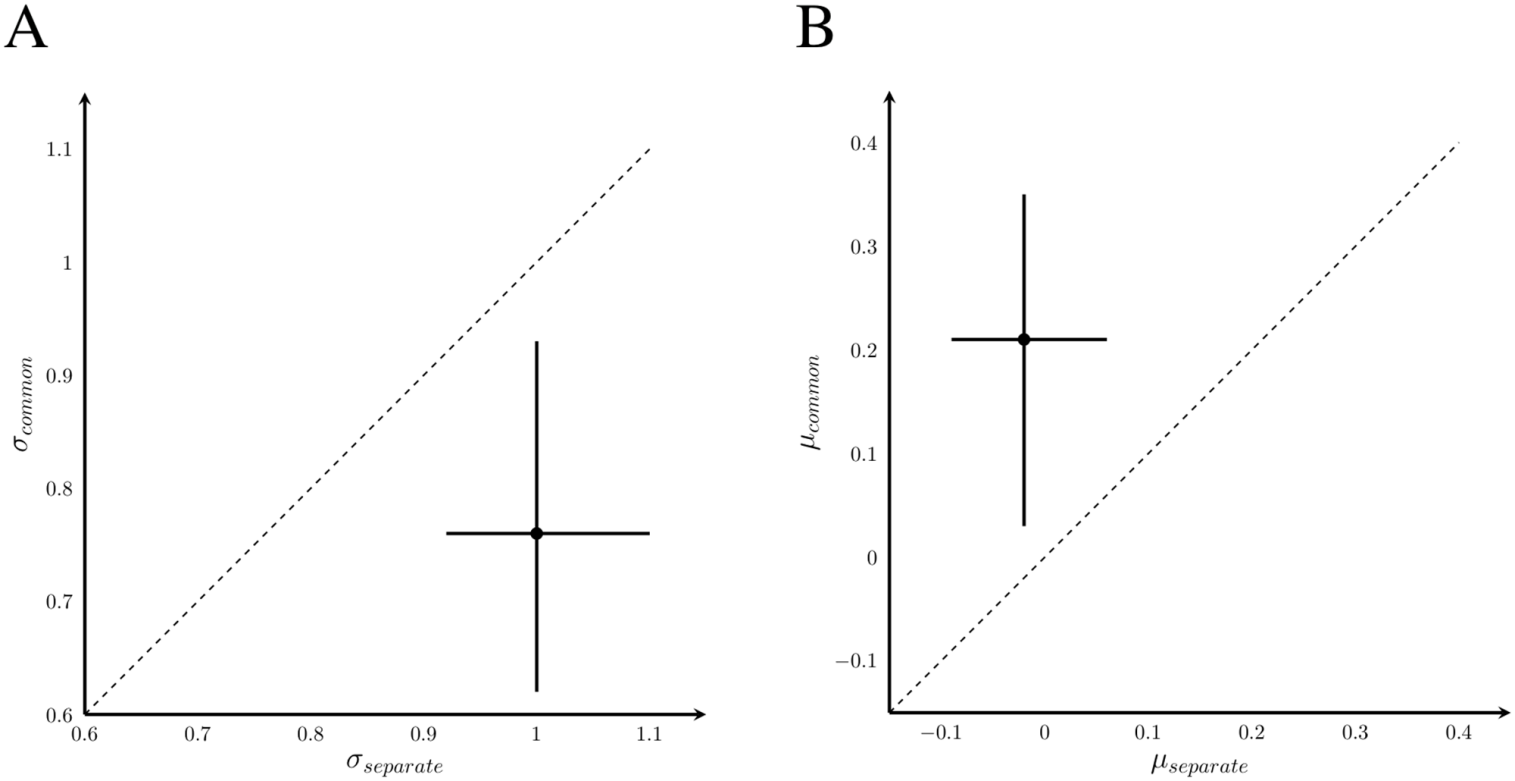
A group analysis of the temporal-conflict condition (Exp 1) split by whether a common separate judgement is more likely (Exp 2). Multisensory trials in the temporal-conflict condition were pooled across subjects using the *z*-transform method, and separated based on whether a “common” source or “separate” sources judgement was more likely according to the GLMM results of Exp 2. A: Rate sensitivity. B: PSE. Error bars: 95% CIs, *N*_*common*_ = 448 trials, *N*_*separate*_ = 1552 trials.

To directly measure how maximum click-flash offset affected rate discrimination in Exp 1, we conducted a group-level analysis of the same style as used for assessing attention-switching and trial-duration effects. Only multisensory trials in the temporal-conflict condition were used for this analysis. As in Exp 1, stimulus levels were transformed into units of SNR (Eq 3) then split by whether a “common source” or “separate sources” judgement was more likely according to the GLMM fit. The maximum-offset boundary between “common” and “separate” was 103 ms, and split the 2000 trials pooled across subjects into 448 high probability of “common” response trials (22.4%) and 1552 “separate” more likely trials (77.6%). Note that all of the simulated sequences of Raposo et al. [20] would be classified as “common” more likely. Independent psychometric functions were then fit, with the PSE and threshold parameter estimates reported in Fig 10.

As can be seen in Fig 10A, the threshold is significantly lower for the psychometric function describing behaviour when a “common” response is more likely. This suggests that rate discrimination is better when the sequence is more likely to be perceived as coming from a common source, because it is more likely to be integrated despite the temporal conflict. There was also a significant effect in the PSE (see Fig 10B), where a higher event rate was required to perceive the comparison stimulus as the same rate as the standard stimulus when “common” was the more likely response. Why were integrated sequences biased in this manner? A simulation showed that audiovisual sequences matching the properties of the standard stimulus have a 17.6% chance of being below the integration boundary of 103 ms, so most are likely perceived as containing separate sources. Thus, integrated audiovisual sequences may appear to have fewer events or a lower density of events than those that are not. Theoretically, only a small bias should be present for comparison sequences perceived as coming from separate sources. While this is a compelling explanation for the observed relative differences in PSE, we unfortunately cannot directly test these hypotheses due to the *z*-transform in our analysis.

## General discussion

In this study we investigated the use of temporal causal-inference cues when observers were presented with stochastic audiovisual sequences in both rate-discrimination and causal-inference experiments. Exp 1 examined multisensory integration in the context of rate discrimination, inspired by the finding that stochastic click-flash sequences were integrated optimally regardless of whether event timing was synchronous or independent [20]. In contradiction with this previous study, our results show that optimal integration is most likely to occur when the auditory and visual signals are both spatially and temporally congruent. Exp 2 explored which temporal features are salient causal-inference cues to understand how different sequence-generating algorithms may have led to conflicting experimental results. Temporal pattern sensitivity and the maximum temporal offset between consecutive clicks and flashes were found to modulate whether sources appeared to share a common origin, whereas the proportion of synchronous click-flash pairs did not. These findings help explain the discrepancy in rate-discrimination results between Exp 1 and Raposo et al. [20]. Specifically, our sequence generating algorithm provided stronger temporal causal-inference cues than those available in the previous study, according to our simulation analysis. Thus our subjects showed less inclination to optimally combine audiovisual information in the face of temporal conflict. As algorithms with reduced causal-inference cues are preferable for cue-conflict studies [21], our results can also be informative for sequence design in such tasks.

How did observers integrate the auditory and visual sequences to form a rate estimate? It is likely that the multisensory mechanism responsible for the observed optimal behaviour integrates rate estimates computed separately for modality rather than individual click-flash events. This is because the clicks and flashes were presented at suprathreshold levels, there is little to gain in terms of detectability of individual events via multisensory integration. Separate rate estimates, however, involve noisy temporal integration, and these estimates could be improved by combining information across the senses. Such a neural mechanism may be present in the superior temporal sulcus, as studies have found auditory and visual temporal pattern sensitivity as well as temporal synchrony processing in this region [46, 47].

Discrimination behaviour at the group level indicated that both counting and density-estimation strategies were used to form rate estimates in Exp 1. The analysis was unable to clarify when each strategy is more likely to be employed, however, other research indicates rate is a likely factor. For example, verbal counting appears to be limited to rates below 6 events/s [48], but non-verbal accumulation strategies associated with rate estimation may not adhere to this low rate limit [49, 50]. Thus, further research is need to determine when count information versus density information is extracted from the encoded temporal patterns.

From our joint analysis of Exps 1 and 2, we also find that perceived audiovisual rate depends on whether the sequences are integrated. In the temporal-conflict condition, sequences likely to be integrated were biased towards lower rate estimates compared to those that were likely to be perceived as containing separate sources. A different strategy may be employed when estimating rate for sequences deemed to be of different causal origins. For an audiovisual sequence perceived as coming from separate sources, a single count or density estimate can be computed by pooling across modalities, and the estimate halved when comparing to a unisensory or integrated multisensory presentation. Conversely, these unisensory or integrated sequences could be doubled for the comparison. Insufficient scaling—applying a factor between 0.5 and 1 instead of halving or between 1 and 2 instead of doubling—would result in discrimination biases like those we observed. Evidence from studies on rhythm perception suggests that audiovisual sequences may be encoded with a modality-independent code at slower rates [51], or automatically converted to an auditory code regardless of rate [52]. A lack of modality-dependent coding is 1) consistent with our findings suggesting combined encoding in the temporal-conflict condition, and 2) likely to facilitate rapid integration of rate estimates in no-conflict scenarios.

How does synchrony affect integration when several clicks and flashes are involved? Paradigms with a single transient audiovisual event emphasise the relative timing of the click and flash, but observers in Exp 2 were faced with a long stochastic sequence of stimulation where the relative timing of each click-flash pair varied from one moment to the next. The results show that maximum click-offset was a salient cue whereas the proportion of synchronous click-flash events was not, indicating that causal-inference judgements were preferentially driven by a temporal mismatch signal. This suggests that memory constraints may contribute to causal-inference strategy for longer periods of audiovisual stimulation. However, our result may not generalise to cases of weaker signals. The maximum click-flash offset only considers a single event pair in a sequence, and is therefore less robust to sensory noise than the proportion of synchronous pairs, which takes into account all events. Thus the relative salience of these cues may depend on the detectability of the individual sensory events.

Although the proportion of synchronous click-flash pairs was not a salient cue, we do find evidence of observers considering properties of the audiovisual sequence on a broad time scale in terms of pattern-similarity sensitivity. In fact, the proportion-synchronous cue is comparable to pattern similarity, but for only a single temporal lag. We should caution here that only click-flash pairs that were coded to occur at the same time were labelled as synchronous, and therefore is likely an inaccurate estimate of the proportion truly perceived as synchronous. Thus, our pattern-similarity analysis was a more sensitive measure, especially since it is well known that the optimal temporal lag for perceived audiovisual synchronicity varies across individuals as well as the width of the audiovisual binding window [16], and these can be affected by rate [53]. Our results indicate that correlations between ±200 ms are more likely to lead to integrated percepts, which is consistent with estimates of thresholds for desynchrony detection with complex speech stimuli [54]. Under dynamic circumstances, the rapid temporal recalibration between the auditory and visual modalities observed previously [55, 56] may interact with temporal causal inference for long sequences of stimulation to maximise sequence correlation in a rate-dependent manner.

It is unlikely that a correlation-detection mechanism assesses pattern similarity at all possible audiovisual lags. Memory constraints would have to impose limits as the number of correlation comparisons grows with the length of the sequence. Exp 2 demonstrates that correlations between ±200 to 800 ms can lead to observers reporting “separate sources” with significantly greater probability, providing an empirical upper limit for lagged correlation comparisons this task. But whether the brain computes this correlation online or performs a post-stimulation comparison of remembered temporal patterns is up for debate. Overall, the results from the pattern similarity analysis demonstrate that dynamic, more complex stimuli contain additional information for making inferences about the state of the world beyond that uncovered by single-event studies.

The findings of the pattern similarity analysis also answer an open question: does the low-pass temporal filtering in the multisensory correlation-detection mechanism proposed by Parise et al. [13] prohibit correlation detection at high temporal rates? Using a nearly identical method, we show that correlation between signals at rates in the range of 8 to 14 events/s can be detected by human observers with a pattern of sensitivity similar to what they observed at 5 events/s. Indeed, even negative portions in the CCG difference functions were found in our task and by Parise et al. [13], with statistical significance confirmed by a permutation analysis in the current study. Thus we do not find evidence of low-pass filtering hampering correlation detection for fast rates. Instead, our results support the stimulus-complexity effects detailed by Denison et al. [27]. Greater inter-stimulus interval variability decreases correlation for temporal-conflict sequences within the ±200 ms temporal window of integration.

Finally, we touch on the topic of spatial conflict in our task. In contrast to recent evidence that spatial congruence is not a major factor for integration in temporal multisensory tasks [32], we observed spatial-conflict sensitivity in the majority of participants. As the spatial relation between the auditory and visual sequences is unchanging within a session, whereas the salient temporal causal-inference cues require some time to ascertain, these robust spatial causal-inference cues may have played a larger role in our non-spatial task than would be expected. If this hypothesis is correct, spatial causal-inference cues should be less salient for rate discrimination if the position of individual events is also stochastically manipulated. Thus, further experimentation is needed to clarify the role of spatial congruency in rate perception.

In sum, the results of our rate-discrimination and causal-inference experiments offer a parsimonious explanation of why optimal audiovisual integration may occur in situations of temporal conflict. Importantly, the temporal causal-inference cues available from stochastic sequences are not just direct extensions of those observed for single-event paradigms and must be considered when selecting sequence-generation algorithms for research. We do see, however, the characteristic trade-off between selectivity for synchronous stimulation and invariance to inter-sensory delays so familiar to multisensory researchers.

## Supporting information

S1 File Experiment 1: Adaptive procedure. (PDF)
