# Correction: Temporal causal inference with stochastic audiovisual sequences

**DOI:** 10.1371/journal.pone.0186922

**Published:** 2017-10-18

**Authors:** Shannon M. Locke, Michael S. Landy

Fig 1 is incorrect. The authors have provided a corrected version here.

**Fig 1:**
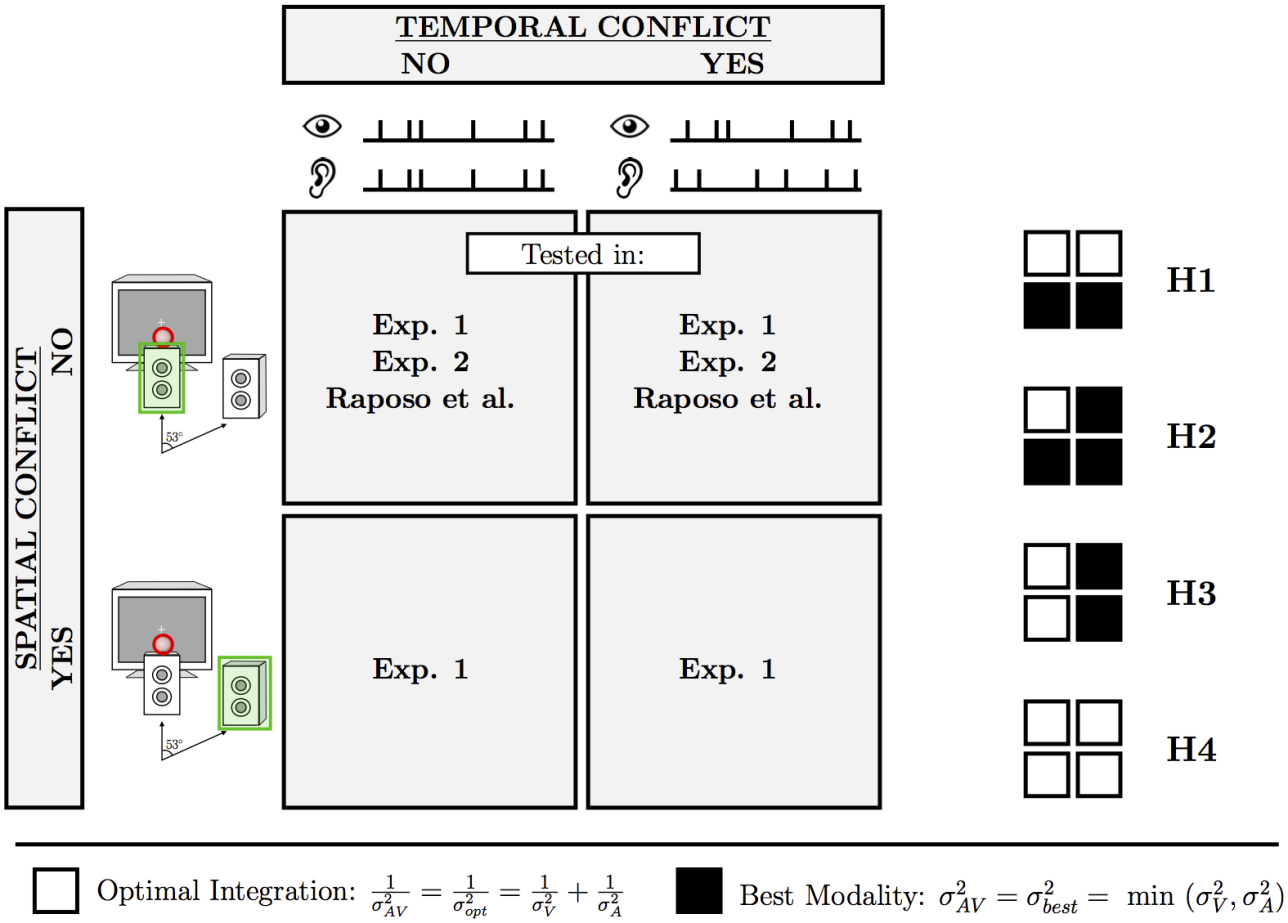
Design and hypotheses of Exp 1. The four spatiotemporal conflict conditions were defined by the spatiotemporal relationship between the auditory and visual signals in Exp 1. Only temporal conflict was examined in Exp 2 and the previous rate-discrimination study of Raposo et al. [20]. The small grids on the right show the predicted pattern of results in Exp 1 under the four hypotheses.
