# Supplementary material for: Temporal causal inference with stochastic audiovisual sequences
Source: PLoS One. 2017 Sep 8;12(9):e0183776. doi: 10.1371/journal.pone.0183776 (PMC5590827; doi:10.1371/journal.pone.0183776)
Supplement: S1 File — (PDF) [file pone.0183776.s001.pdf]

Temporal Causal Inference With Stochastic Audiovisual Sequences

Shannon M. Locke[1*], Michael S. Landy[1,2]

1 Dept. of Psychology, New York University, New York, NY, United States

2 Center for Neural Science, New York University, New York, NY, United States

Supporting Information

S1 Appendix. Experiment 1: Adaptive Procedure. The adaptive procedure used in Exp 1 was a modified version of the updated Maximum-Likelihood method from the UML toolbox [1]. The algorithm fits a psychometric function of chosen form after each trial given all previous stimulus levels and responses, and then estimates the 'sweet points'. These are the optimal sampling points on the true underlying function, which give parameter estimates with the lowest expected variance [1,2]. In the original algorithm, subsequent stimulus levels are selected from these sweet points in a staircase-like manner.

The response metric in our task was the proportion of comparison stimuli judged faster than the 8 events/s standard. We fit a cumulative Gaussian distribution after each trial: $P(\text{comparison judged faster}) = \lambda + (1 - 2\lambda)\Phi(\mu, \sigma)$. The first five participants, however, were tested using the following incorrect form of this function $P(\text{comparison judged faster}) = (1 - \lambda)\Phi(\mu, \sigma)$. The staircase component of the UML procedure requires psychometric functions to have the lower asymptote be the guess rate, but our function instead ranged from 0 to 1. To choose the next stimulus intensity, we selected a μ, σ, and λ sweet point with 30%, 60%, and 10% probability respectively. More trials were devoted to sampling the σ sweet points because optimality was assessed with this parameter. Flat priors were used for the μ, σ, and λ parameters, with the ranges set based on the results of pilot data (μ: 100

linearly-spaced values [5, 11]; σ 60 linearly-spaced values [0.3, 3.5]; and λ 10 linearly-spaced values [0, 0.1]).

References

1. Shen Y, Richards VM. A maximum-likelihood procedure for estimating psychometric functions: Thresholds, slopes, and lapses of attention. Journal of the Acoustical Society of America. 2012;132(2):957–967.

2. Green DM. Stimulus selection in adaptive psychophysical procedures. Journal of the Acoustical Society of America. 1990;87(6):2662–2674.
